# Magnetic Resonance Cholangiopancreatography (MRCP) Findings in a Patient With AIDS Cholangiopathy and Cryptosporidiosis

**DOI:** 10.7759/cureus.45869

**Published:** 2023-09-24

**Authors:** Rasheed Abdullah, Mustafa Azam, Desiree Clement, Sayf Al-Katib

**Affiliations:** 1 Radiology, Oakland University William Beaumont School of Medicine, Rochester, USA; 2 Diagnostic Radiology and Molecular Imaging, Corewell Health William Beaumont University Hospital, Royal Oak, USA

**Keywords:** mrcp, ercp, biliary stricture, cryptosporidiosis, hiv, aids cholangiopathy

## Abstract

AIDS cholangiopathy is a rare condition characterized by intra- and extra-hepatic ductal strictures causing biliary obstruction primarily in individuals with advanced HIV infection and low clusters of differentiation 4 (CD4) count. This case report presents a male patient with a history of HIV, poor adherence to antiretroviral therapy (ART), and chronic cryptosporidiosis infection, who exhibited clinical and radiological findings consistent with advanced immunocompromise and AIDS cholangiopathy. The patient presented with respiratory symptoms, weight loss, renal dysfunction, and elevated liver enzymes. Imaging studies, including ultrasound and magnetic resonance cholangiopancreatography (MRCP), revealed diffuse biliary dilatation and stricturing, indicative of cholangiopathy. Subsequent endoscopic retrograde cholangiopancreatography (ERCP) with stent placement was performed to manage the biliary obstruction. This case highlights the importance of considering AIDS cholangiopathy in HIV patients with poor ART compliance who present with biliary obstruction symptoms or cholestatic liver enzyme abnormalities. Prompt diagnostic evaluation using MRCP or ERCP can aid in confirming the diagnosis and guiding appropriate therapeutic interventions, including endoscopic management and initiation of ART.

## Introduction

AIDS cholangiopathy is a sclerosing cholangitis characterized by strictures causing biliary obstruction in the setting of chronic infection among those with markedly reduced clusters of differentiation 4 (CD4) count. The typical presentation includes right upper quadrant pain, diarrhea, nausea, and vomiting, although it can also be asymptomatic [1]. The incidence of this pathology has markedly decreased since the introduction of effective antiretroviral therapy (ART) [2,3]. Here, we present a case of a male with a history of HIV and poor compliance with ART who was found to have magnetic resonance cholangiopancreatography (MRCP) findings of AIDS cholangiopathy in the setting of a chronic cryptosporidiosis infection.

## Case presentation

A 36-year-old male with a past medical history of HIV presented to the emergency department with cough, dyspnea, fatigue, and productive green sputum for the past week. He also reports a recent history of diarrhea and decreased appetite leading to a 17-pound weight loss over the past seven months. He was diagnosed with HIV two years ago by his primary care physician (PCP) and placed on a regimen consisting of abacavir/dolutegravir/lamivudine; however, the patient had been poorly adherent claiming he takes them “a few days a week.” He also reported a prior history of poorly described "kidney problems" that required urgent hemodialysis in the past.

In the emergency department (ED), he was ill-appearing and cachectic. He was hypotensive with a blood pressure of 89/50 mmHg. His remaining vital signs were within normal limits but his body mass index (BMI) was 12.8. His mucous membranes were dry and his abdomen was soft, nondistended, and non-tender. The remaining physical exam was unremarkable. A chest x-ray and non-contrast computed tomography (CT) study of the head were both normal-appearing. Laboratory results were significant for impaired renal function with a creatinine of 5.99 mg/dL and blood urea nitrogen (BUN) of 65 mg/dL. This likely represented an acute kidney injury (AKI) on possible chronic kidney disease (CKD) given the patient’s prior kidney dysfunction, although the patient's previous baseline creatinine is unknown. The patient was placed on urgent hemodialysis. He also had mild anemia with a hemoglobin of 10.1 g/dL. His white blood cell (WBC) count was within normal limits however his CD4 was 18 cells/mm^3 and his HIV viral load was 50. He had elevated liver enzymes in a cholestatic pattern with bilirubin 3.2 mg/dL, alkaline phosphatase (ALP) 650 U/L, aspartate aminotransferase (AST) 120 U/L, and alanine transaminase (ALT) 148 U/L. An acute viral hepatitis panel was non-reactive, influenza and COVID testing were negative, and a subsequent kidney, ureter, and bladder (KUB) X-ray was normal. Blood cultures were collected and infectious diseases (ID) were consulted. They placed the patient on a regimen of trimethoprim/sulfamethoxazole, azithromycin, cefepime, and vancomycin. Non-contrast CT of the chest was performed and showed bilateral ground-glass opacities and left pleural effusion. Sputum stain for *Pneumocystis jirovecii* was negative. The patient declined further diagnostic evaluations for respiratory symptoms including bronchoscopy and bronchoalveolar lavage so ID resolved to treat prophylactically for *Pneumocystis* pneumonia. Diagnostic workup for diarrhea included stool cultures which detected *Cryptosporidium parvum* and the patient was begun on nitazoxanide.

**Figure 1:**
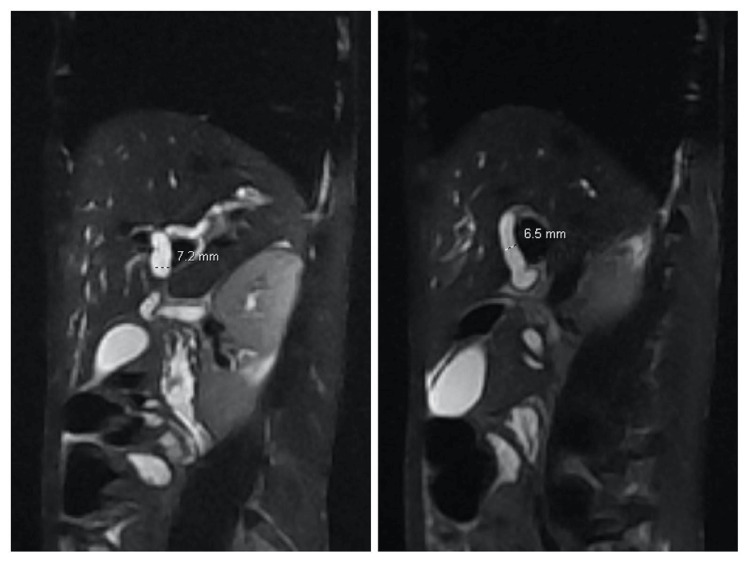
The proximal left (left image) and right (right image) hepatic ducts are dilated, measuring approximately 7.2 mm and 6.5 mm, respectively.

**Figure 2:**
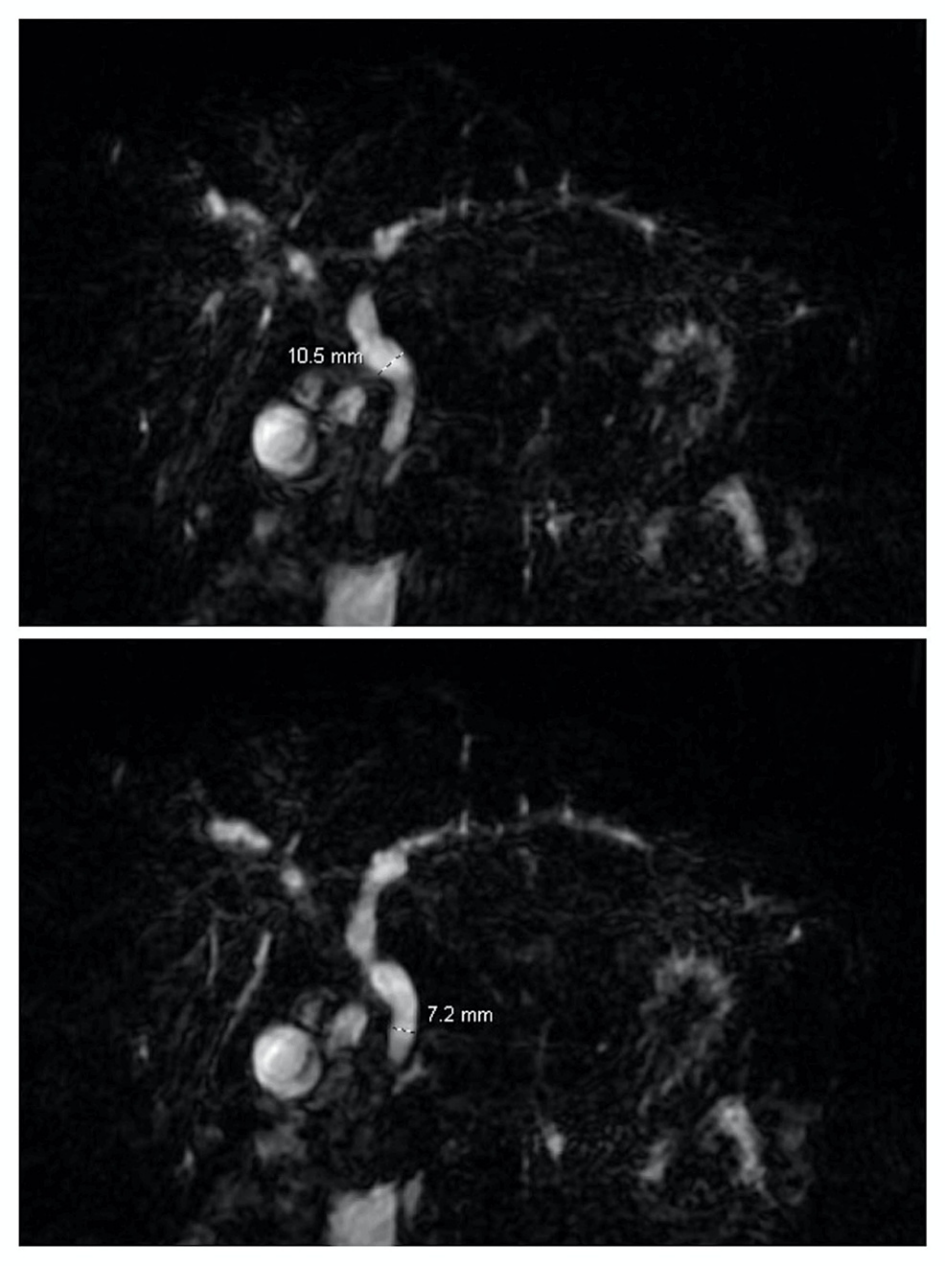
The common bile duct measures 10.5 mm proximally (top image), and 7.2 mm distally (bottom image). The common bile duct demonstrates a relatively smooth, tapering appearance to the ampulla of Vater.

**Figure 3:**
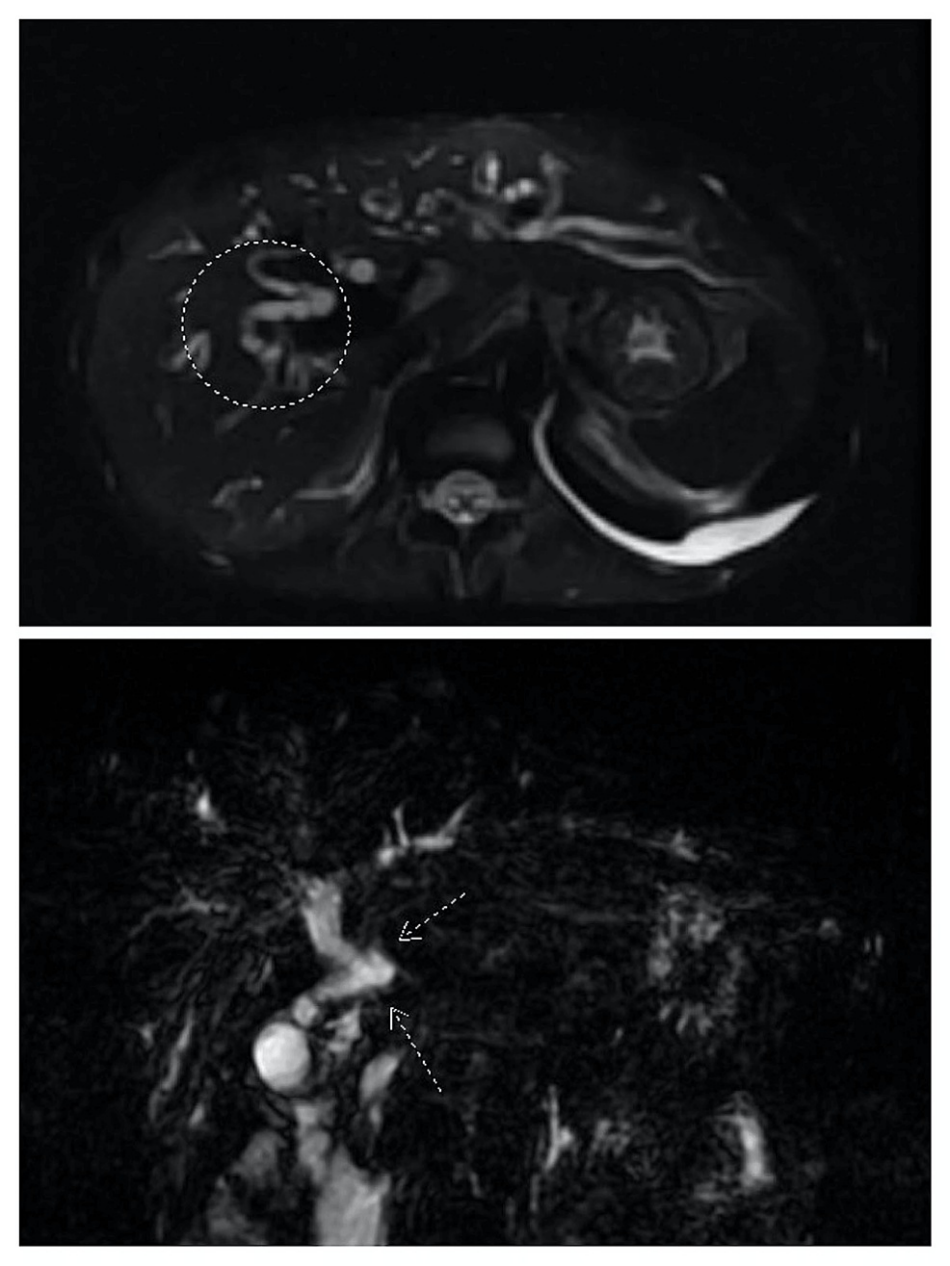
There is variant biliary anatomy, with a high insertion of the cystic duct at the confluence of the right and left hepatic ducts (circled in top image). The intrahepatic biliary tree is mildly irregular in appearance, with several subtle areas of undulation with intervening mild dilatation (as shown by arrows in bottom image).

**Figure 4:**
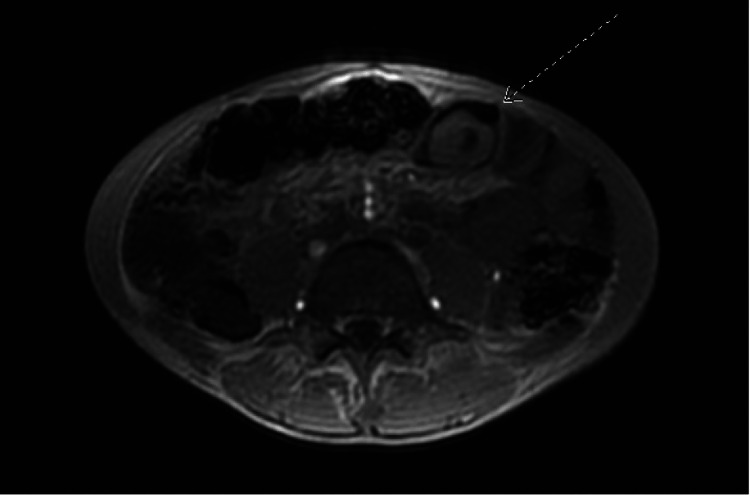
At least two areas of small bowel-small bowel intussusception were identified (arrow points to only one area).

**Figure 5:**
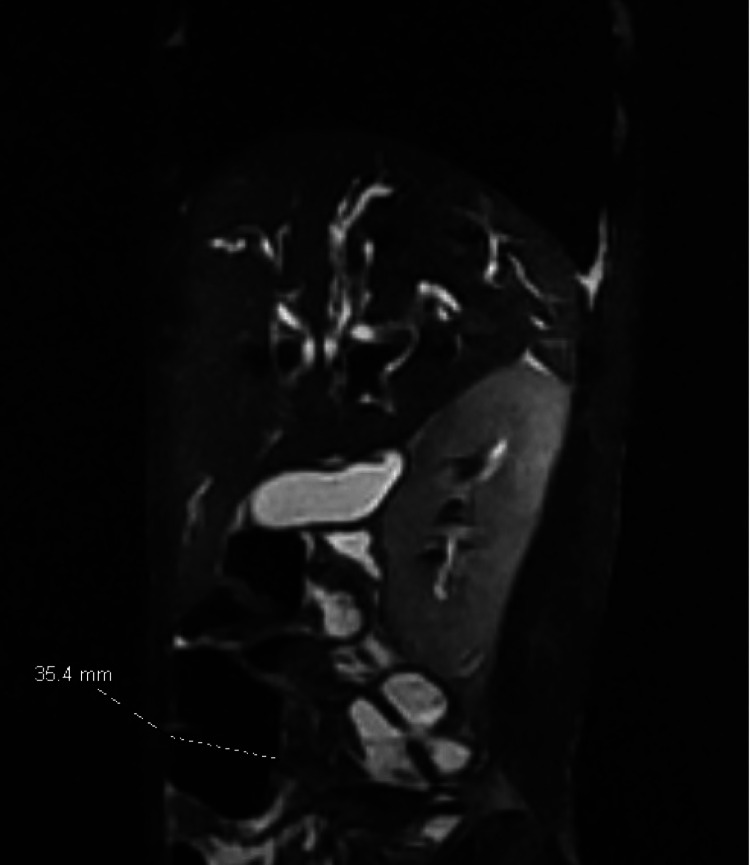
Mild dilatation of several loops of small bowel measuring up to 35.4 mm.

**Figure 6:**
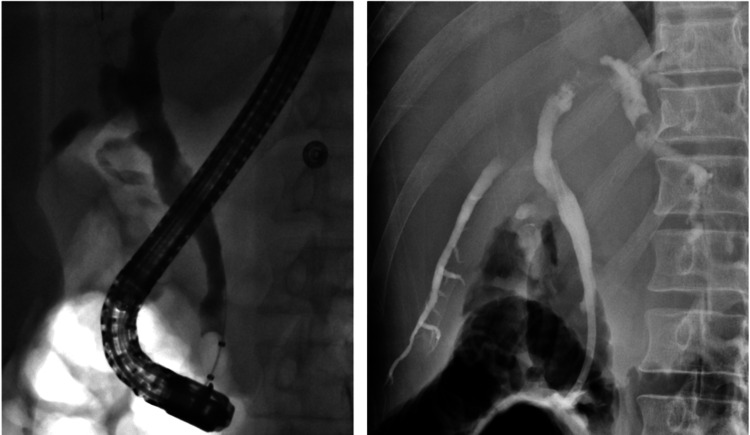
ERCP procedure showing placement of endoscope and catheter (left image) and contrast instillation into the biliary tree (right image).

The patient’s elevated liver enzymes prompted ultrasonography (US) of the abdomen which revealed intra- and extrahepatic biliary dilatation with the common bile duct measuring 9 mm. A subsequent MRCP further revealed diffuse dilatation of the intra- and extrahepatic biliary tree without evidence of an obstructing mass or stone (Figure 1). The common bile duct (CBD) was also dilated with measurements consistent with the earlier US study (Figure 2). There appeared to be subtle, undulating irregularity to the intrahepatic biliary tree, with focal areas of mild stricturing and intervening dilatation (Figure 3). Given the prominent extrahepatic dilatation, an underlying stenosis at the duodenal papilla was likely present. Additional MRCP findings included small bowel-small bowel intussusception in at least two areas that persisted throughout the exam (Figure 4) as well as mild diffuse small bowel dilatation (Figure 5). The findings of intussusception and biliary findings were thought to be the sequela of the patient's chronic cryptosporidium infection and HIV cholangiopathy, respectively. A CT enterography was recommended to evaluate the findings of intussusception; however, the patient declined the procedure. General surgery was consulted and determined the patient was stable for discharge given at the time he was able to eat, was having regular bowel movements, and was passing flatus as well as having an overall lack of concerning symptoms. He was discharged with plans to undergo CT enterography within a month. Three months after the patient’s ED presentation, he underwent ERCP (Figure 6) to treat his cholangiopathy. ERCP demonstrated filling defects in the CBD and the left main hepatic duct that was presumed to be sludge. Diffuse nodular mucosa was noted throughout the first and second portions of the duodenum. Biopsies taken from the duodenum and bile duct at the ampulla were unremarkable. A 5 mm biliary sphincterotomy was made and a 10 Fr by 7 cm plastic stent was placed in the CBD. Upon placement of the stent, good bile flow was observed, and the patient was sent back to the hospital ward for recovery.

## Discussion

*Cryptosporidium parvum* is an opportunistic parasite that can lead to a severe gastrointestinal infection among those with AIDS or an immunocompromised status [4]. It typically presents with watery diarrhea and dehydration and in the case of a prolonged chronic course, it can lead to the development of an acalculous obstruction in the biliary ducts characterized by intra-and extra-hepatic strictures and dilatation [1]. This pattern of stricturing leading to biliary obstruction defines AIDS cholangiopathy. Our patient with AIDS cholangiopathy likely contracted a cryptosporidium infection at least seven months prior to his ED presentation, given his weight loss history. It is important to note that AIDS cholangiopathy can develop in the setting of other infections including cytomegalovirus (CMV), microsporidium, mycobacterium avium complex (MAC), and herpes simplex virus (HSV). Moreover, in up to 50% of patients, no definite organism source is identified [3]. Additionally, while AIDS cholangiopathy was the leading diagnosis for our patient, autoimmune conditions could not be ruled out definitively at the time. AIDS cholangiopathy has a variable presentation, but typically involves right upper quadrant pain, diarrhea, nausea, and vomiting, although it can also be asymptomatic. Laboratory abnormalities commonly demonstrate elevated liver enzymes in a cholestatic pattern [2]. The combination of gastrointestinal symptoms and elevated liver enzymes in patients poorly compliant with ART, particularly those with a CD4 count < 100 cells/microL [5,6] should prompt clinicians to investigate for changes associated with AIDS cholangiopathy.

In patients with AIDS cholangiopathy, the initial imaging evaluation consists of ultrasonography followed by MRCP or ERCP [7]. These studies may demonstrate one of four various patterns. The most common is a combination of sclerosing cholangitis and papillary stenosis (50%). Our patient’s imaging findings revealed a pattern most consistent with this variation. Additional variations include an isolated intrahepatic sclerosing cholangitis-like appearance (20%), an isolated papillary stenosis (15%), or a long-segment extrahepatic duct stricture with or without an associated intrahepatic disease (15%) [3,8]. The therapeutic management of AIDS cholangiopathy primarily involves ERCP. In patients with underlying papillary stenosis causing symptoms of biliary obstruction, ERCP with biliary sphincterotomy can provide symptomatic relief [9,10]. In patients who instead have biliary stricturing in the CBD, symptomatic relief is provided by ERCP with stent placement [11]. Our patient underwent a biliary sphincterotomy for papillary stenosis and stent placement as both abnormalities were present. Alongside endoscopic management for biliary obstruction, patients should be placed on an antimicrobial for any associated infection and all patients should be placed on ART as soon as possible.

This case report describes a patient with a documented history of HIV infection, a CD4 count of less than 100 cells/microL, and a cholestatic pattern of liver enzymes. MRCP revealed multifocal strictures and segmental dilatations in the biliary system, leading to a diagnosis of AIDS cholangiopathy [12]. However, it is important to consider that cryptosporidium infection can also directly affect the biliary system independently of AIDS cholangiopathy. Biliary cryptosporidiosis exhibits similar clinical presentations to AIDS cholangiopathy, including complications such as intrahepatic bile duct strictures and dilatation of the common hepatic and common bile ducts [13]. Despite the probable diagnosis of AIDS cholangiopathy based on established diagnostic criteria, biliary cryptosporidiosis could not be definitively ruled out due to the absence of specific diagnostic testing for this infection in the biliary system. Further investigations would be warranted to distinguish between these two disease processes in immunocompromised individuals. The presence of small bowel-small bowel intussusception has previously been described in patients with cryptosporidiosis [14].

## Conclusions

AIDS cholangiopathy should be highly suspected in patients with HIV who are not compliant with ART and present with symptoms of biliary obstruction or lab abnormalities consistent with cholestasis. Diagnostic evaluation can include MRCP or ERCP to visualize intra- and extrahepatic duct strictures with associated dilatation. The treatment for AIDS cholangiopathy involves ERCP with stenting or biliary sphincterotomy, depending on the type of stenosis present. 
